# Analysis of Pyrrolizidine Alkaloids in Queensland Honey: Using Low Temperature Chromatography to Resolve Stereoisomers and Identify Botanical Sources by UHPLC-MS/MS

**DOI:** 10.3390/toxins11120726

**Published:** 2019-12-11

**Authors:** Natasha L. Hungerford, Steve J. Carter, Shalona R. Anuj, Benjamin L. L. Tan, Darina Hnatko, Christopher L. Martin, Elipsha Sharma, Mukan Yin, Thao T. P. Nguyen, Kevin J. Melksham, Mary T. Fletcher

**Affiliations:** 1 Queensland Alliance for Agriculture and Food Innovation (QAAFI), The University of Queensland, Brisbane, QLD 4072, Australia; christopherlancemartin@gmail.com (C.L.M.); e.sharma@uq.net.au (E.S.); mukan.yin@uq.net.au (M.Y.); tran.nguyen7@uq.net.au (T.T.P.N.); 2 Forensic and Scientific Services, Queensland Health, Brisbane, QLD 4108, Australia; steve.carter@health.qld.gov.au (S.J.C.); shalona.anuj@health.qld.gov.au (S.R.A.); benjamin.tan@health.qld.gov.au (B.L.L.T.); Darina.Hnatko@health.qld.gov.au (D.H.); Kevin.Melksham@health.qld.gov.au (K.J.M.)

**Keywords:** LC-MS, pyrrolizidine alkaloid, honey, *Parsonsia straminea*, lycopsamine, indicine, *Heliotropium amplexicaule*

## Abstract

Pyrrolizidine alkaloids (PAs) are a diverse group of plant secondary metabolites with known varied toxicity. Consumption of 1,2-unsaturated PAs has been linked to acute and chronic liver damage, carcinogenicity and death, in livestock and humans, making their presence in food of concern to food regulators in Australia and internationally. In this survey, honey samples sourced from markets and shops in Queensland (Australia), were analysed by high-resolution Orbitrap UHPLC-MS/MS for 30 common PAs. Relationships between the occurrence of pyrrolizidine alkaloids and the botanical origin of the honey are essential as pyrrolizidine alkaloid contamination at up to 3300 ng/g were detected. In this study, the predominant alkaloids detected were isomeric PAs, lycopsamine, indicine and intermedine, exhibiting identical MS/MS spectra, along with lesser amounts of each of their *N*-oxides. Crucially, chromatographic UHPLC conditions were optimised by operation at low temperature (5 °C) to resolve these key isomeric PAs. Such separation of these isomers by UHPLC, enabled the relative proportions of these PAs present in honey to be compared to alkaloid levels in suspect source plants. Overall plant pyrrolizidine alkaloid profiles were compared to those found in honey samples to help identify the most important plants responsible for honey contamination. The native Australian vines of *Parsonsia* spp. are proposed as a likely contributor to high levels of lycopsamine in many of the honeys surveyed. Botanical origin information such as this, gained via low temperature chromatographic resolution of isomeric PAs, will be very valuable in identifying region of origin for honey samples.

## 1. Introduction

Pyrrolizidine alkaloids (PAs) are secondary metabolites that comprise more than 600 compounds, typified by a pyrrolizidine base with one or more ester linkages. The 1,2-unsaturated PAs are toxic to animals and humans, causing acute and chronic liver and lung damage or cancer and are mainly produced by flowering plant species belonging to the families Asteracaeae (Compositae, tribes Senecioneae and Eupatorieae), Fabaceae (Crotolaria, Chromolaena, Lotonis), Apocynaceae (Echiteae) and Boraginaceae [1], estimated to represent 3% of the Earth’s flowering plants [2]. Plants containing pyrrolizidine alkaloids are globally distributed and PAs provide a chemical defence for plants against herbivores. PA biosynthesis has been found to be dependent on many factors, with implications for plant/animal/insect interactions [3]. Various food products can contain toxic PAs either directly from plant origin (certain herbs, herbal medicines) or indirectly through natural transfer from floral nectar and pollen (e.g., some honey, pollen dietary supplements) or inadvertent cross-contamination (e.g., grains, herbs, teas) [4]. The Australian provisional tolerable daily intake of pyrrolizidine alkaloids is 1 µg/kg Bw/day [5], whilst the recommended European accepted intake is 150 times lower at 0.007 µg/kg Bw/day [6,7,8], although this has been recently revised [9,10].

Several studies have described the presence of toxic pyrrolizidine alkaloids (PAs) in honey produced by *Apis mellifera*. Internationally it has been reported that such toxins can be found in honey due to transfer by bees of pollen/nectar from certain flowers, including *Heliotropium*, *Crotolaria*, *Echium* and *Senecio* species. Concern has been raised as to the extent of contamination in Australian honey [11,12] but their presence in Queensland honey has not previously been examined. Previously, investigations into the presence of PAs in Australian honey have concentrated on the introduced pest plant *Echium plantagineum* L. (Paterson’s curse) as the major likely PA source [11,12,13]. However, rigorous eradication and biological control programs in recent decades have decreased the prevalence of this weed in Australia [14]. Diverse PA containing plant species occur in Australian pastures and have intoxicated grazing horses, cattle, sheep or pigs and poultry fed contaminated grains [15,16] and include both native and introduced *Crotalaria, Heliotopium* and *Senecio* species [17]. Additionally, native Australian *Parsonsia* species grow in rainforests and on the margins of rainforest/eucalypt forest and contain PAs known to be sequestered by butterflies [18,19,20]. *Parsonsia* species spread from tropical and subtropical Asia to Australia and the south-west Pacific. *Parsonsia straminea* is native to Queensland and New South Wales [21] but there have been no field reports of livestock poisonings [17]. The distribution of these PA containing plants varies throughout the country and different alkaloids would be expected in honey from tropical/sub-tropical Queensland regions as compared to honey originating from southern temperate states. Given that honey represents a significant food source of human exposure to PAs [10], identification of plant PA sources to reduce this exposure is crucial.

In this study of market honey, samples have been identified with alkaloid profiles that appeared to be consistent with a number of PA containing plant species present within the Australian environment. This study examines the presence of PAs in a market survey of honey purchased in Queensland with the aim to assess any food safety concern for the consumer of honey and to correlate PAs identified with previously unsuspected plant sources of these alkaloids.

## 2. Results and Discussion

### 2.1. Pyrrolizidine Analysis Method Validation

Pyrrolizidine alkaloid levels in honey/plant material were quantitated by HRAM UHPLC-MS/MS analysis against 30 certified PA standards, through comparison of the precursor parent ion intensity (Table 1) to the standard curves, with squared correlation coefficients (R^2^) typically in the range of 0.9932–0.9997. The honey analysis method was validated according to the National Association of Testing Authorities (NATA) guidance document [22]. The method was validated in blank honey, based on results for 10 spiked samples, giving Limits of Reporting (LORs) of 5 ng/g for individual PAs (Table 2). The uncertainties given are at the 95% confidence level as required by NATA [22,23]. Note that for multiresidue analyses at these levels, a default standard uncertainty of ±25% RSD at the 95% confidence level is routinely applied by the authors and is used unless there is evidence that the actual uncertainty is greater than this value. Erucifoline, erucifoline *N*-oxide, jacobine *N*-oxide and seneciphylline *N*-oxide consistently gave low recoveries, resulting in high calculated standard uncertainty (% RSD) for these PAs, but which are not unusual for analyses at these levels. The EU recommends ±50% RSD unless it is demonstrated to be a bigger value [24]. The uncertainty values are calculated at the LOR because it is expected that this level will be the worst case scenario. A small peak was present in the blank for trichodesmine, explaining the higher LOD/LOR and increased uncertainty for this compound.

### 2.2. Alkaloid Levels Measured in Honey

Honeys purchased in supermarkets, health food shops, and from individual commercial/small-scale producers were analysed and calculated to contain pyrrolizidine alkaloids levels between <LOR (i.e., below limit of reporting) to ≈3300 ng/g of honey. 

**Figure 1:**
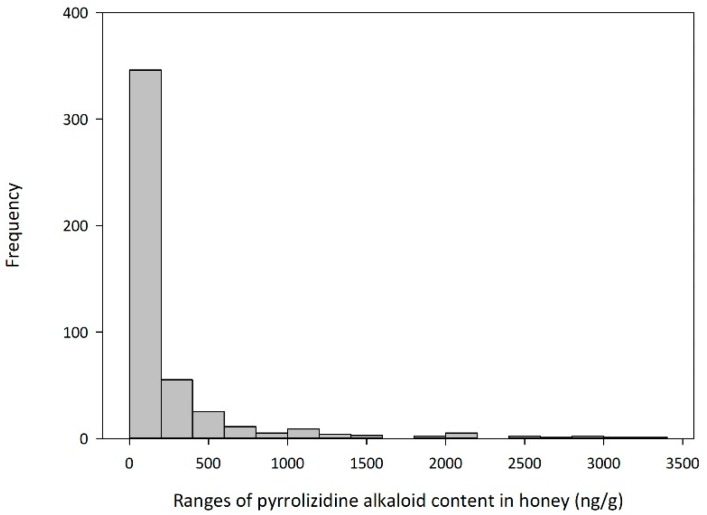
Histogram showing the frequency of total pyrrolizidine alkaloid concentrations in honey samples (*n* = 465) analysed against all 30 pyrrolizidine alkaloid standards (and isolated helioamplexine [25]).

Figure 1 summarises the results in a histogram, with single PA test results below the limit of reporting (5 ng/g) set equal to zero. PAs were detected in 84% of the honey samples examined (*n* = 465). Notably the mean total PA level of PA-positive samples (280 ng/g) was greater than the median (97 ng/g), indicating that the distribution was skewed, with a prevalence of low values (Figure 1, histogram). Whilst the prevalence of low values is reassuring, the overall distribution of total PA concentration is wide, ranging from <LOR to ≈3300 ng/g.

### 2.3. LC-MS/MS Separation of Alkaloids

**Figure 2:**
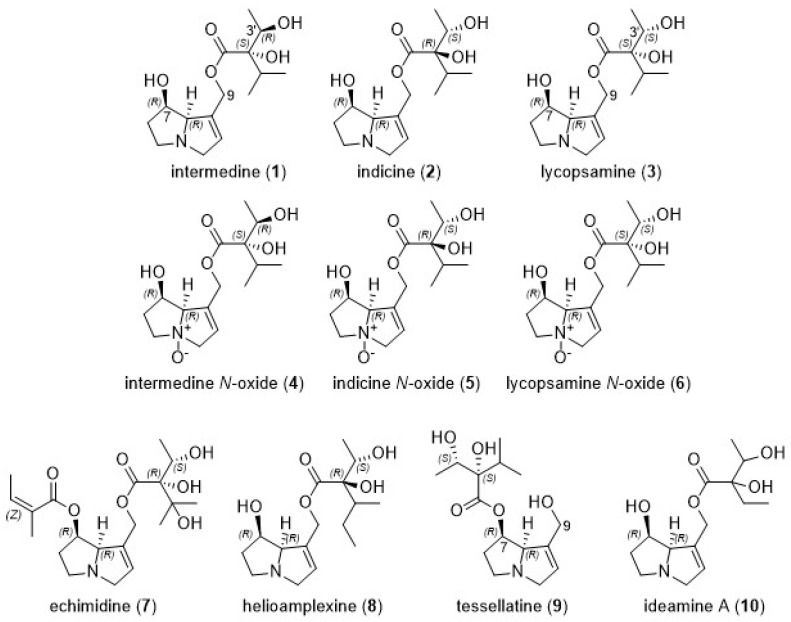
Structures of predominant PAs (**1**–**8**) observed in honey (*n* = 465), together with structures of minor components tentatively observed in *Parsonsia straminea* (**9**–**10**).

**Figure 3:**
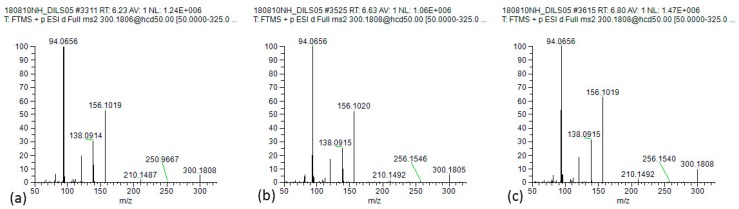
Stereoisomeric pyrrolizidine alkaloids (**a**) intermedine (**1**), (**b**) indicine (**2**), and (**c**) lycopsamine (**3**), with identical high resolution accurate mass spectra.

Analysis revealed that the individual PA pattern detected by the LC-MS/MS analysis of honeys was characterised almost exclusively by lycopsamine-type PAs. In this study the lycopsamine-type PAs were represented by standards intermedine (**1**), indicine (**2**) and lycopsamine (**3**) (Figure 2). These diastereomeric PAs cannot be distinguished based on their MS/MS spectra [26], and Figure 3 shows the identical mass spectra obtained for standards intermedine (**1**), indicine (**2**) and lycopsamine (**3**) by our described HRAMS method. Given the diastereomeric nature of these alkaloids all parent MH^+^ ions and fragment ions are identical, even with HRAMS. Separation based on retention time (RT) was therefore necessary in order to ascertain the botanical origin of PA contamination in these honeys. In most previous studies of PAs in honey, lycopsamine-type PAs were reported as the sum of unresolved stereoisomers, (including indicine (**2**), intermedine (**1**) and/or lycopsamine (**3**), and even the less common rinderine and echinatine) [27,28,29,30] or partially resolved stereoisomers [8,31,32,33]. Under our initial UHPLC conditions, with a column oven temperature of 40 °C, intermedine (**1**) eluted separately first, but indicine (**2**) and lycopsamine (**3**) co-eluted from the Kinetex XB-C18 UHPLC column. Notably the combined indicine/lycopsamine (**2**/**3**) peak represented 75% of the alkaloids present in Queensland honey. As these two alkaloids originate from distinctly different PA plant sources, our aim was to be able to separately quantify the levels of each of these PAs in honey to enable the major plant source of PA contamination to be identified.

**Figure 4:**
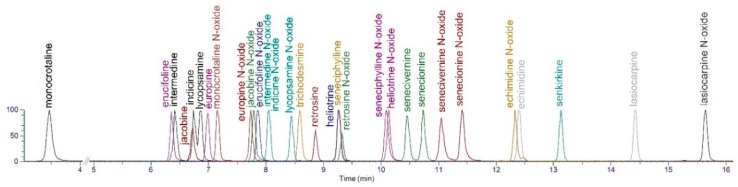
Extracted ion chromatogram of the 30 pyrrolizidine alkaloid calibration standards, illustrating the separation obtained under the UHPLC method, with column temperature of 5 °C.

Ultimately, separation of indicine/lycopsamine (**2**/**3**) was achieved by simply adjusting the column temperature to 5 °C. A more complicated ‘multiple heart-cutting two dimensional chromatography’ method has previously been reported for the resolution of multiple PA isomer pairs [34], but in our hands the simple gradient elution at 5 °C was sufficient to achieve our desired resolution of indicine/lycopsamine (**2**/**3**). Under these conditions, of the 30 PAs and PA-NOs all were resolved based on retention time or mass fragmentation of the MS/MS except for intermedine *N*-oxide (**4**) and indicine *N*-oxide (**5**) which displayed identical RT and MS/MS (Figure 4). In plants where these *N*-oxides (**4**) and (**5**) are prevalent, the *N*-oxides could be distinguished by reduction to the corresponding parent alkaloid (**2**/**3**) which were resolved by RT under the described conditions.

### 2.4. Predominant Alkaloids Present in Queensland Honeys

**Figure 5:**
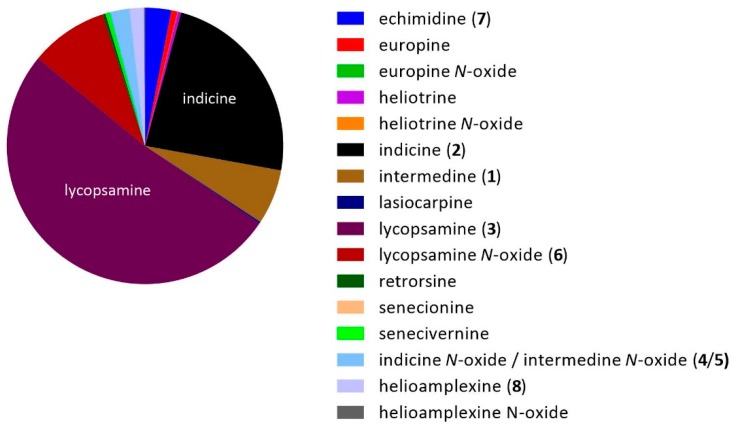
Total amount of each pyrrolizidine alkaloid detected against the 30 PA standards.

Analysis of all 465 honeys under our optimised LC-MS/MS conditions revealed that the predominant pyrrolizidine alkaloid present in our Queensland honey samples was lycopsamine (**3**), which represented approximately 51% of the measured alkaloid content, followed by indicine (**2**) at 24%, lycopsamine *N*-oxide (**6**) at 9%, intermedine (**1**) at 6% and echimidine (**7**) at 3% (Figure 5). Even though we did not resolve intermedine *N*-oxide (**4**) and indicine *N*-oxide (**5**), the identity of the minor *N*-oxide in individual honey samples was inferred by the presence of the co-occurring parent alkaloid (either intermedine (**1**) or indicine (**2**)).

In individual honeys, lycopsamine (**3**) was detected at up to ≈3100 ng/g, indicine (**2**) at up to 1700 ng/g, with the highest total PA content in any individual honey of ≈3300 ng/g which contained mainly a mixture of lycopsamine (**3**) and lycopsamine *N*-oxide (**6**).

**Figure 6:**
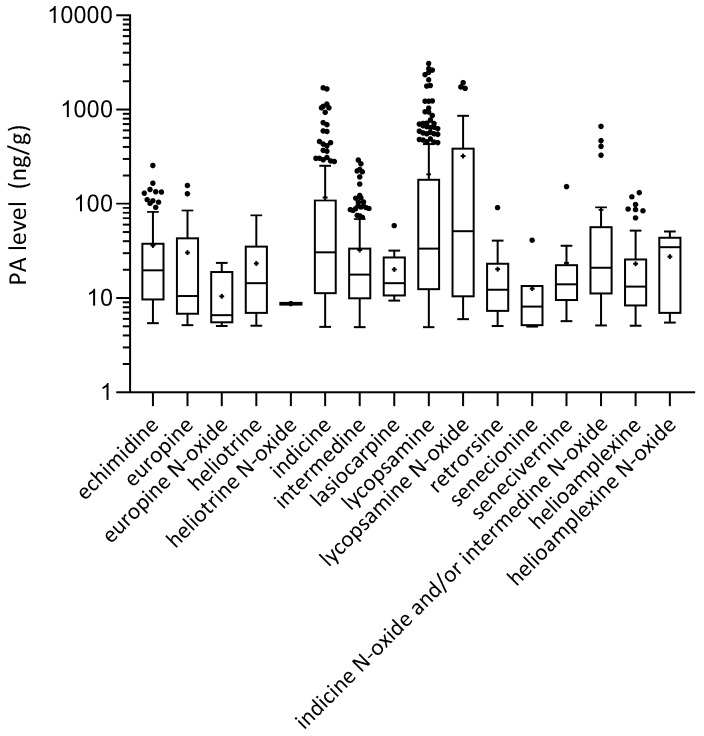
Tukey box and whisker plot of distribution of each pyrrolizidine alkaloid detected in honey (*n* = 465) (includes results >5 ng/g only).

Figure 6 shows a Tukey box and whisker plot of the pyrrolizidine alkaloids detected in honeys (*n* = 465), showing the distribution of each PA concentration, for positive samples only. The largest variation was observed for lycopsamine (**3**), indicine (**2**) and lycopsamine *N*-oxide (**6**). In honeys where lycopsamine (**3**) and its *N*-oxide (**6**) were abundant these were generally the dominant PAs (>90% of PAs detected). 

Similarly, in honeys where indicine (**2**) and its *N*-oxide (**5**) were abundant these were generally the dominant PAs (>68% of PAs detected). In order to explain the relative predominance of these diastereomeric PAs in different honeys, it was clear that we had to identify two main and distinctly different PA plant sources.

### 2.5. Plant Sources of Indicine *(**2**)* in Honey

An examination of the locally abundant weed *Heliotropium amplexicaule* (Blue heliotrope) by our LC-MS/MS method revealed that indicine (**2**) and indicine *N*-oxide (**5**) were the predominant pyrrolizidine alkaloids in this plant, and examination of more minor components including the newly identified helioamplexine (**8**) provided a unique fingerprint in the HRAM LC-MS/MS profile [25]. Interrogation of the pyrrolizidine alkaloid profile from market honey samples with high amounts of indicine (**2**), demonstrated that there was strong correlation between the honey PA profile and the *H. amplexicaule* plant alkaloid profile. The presence of both major and minor *H. amplexicaule* alkaloids in this honey provided strong evidence that this plant represented the floral source for this alkaloid contamination [25].

### 2.6. Plant Sources of Lycopsamine *(**3**)* in Honey

We similarly sought to understand the source of lycopsamine (**3**) (and its *N*-oxide (**6**)), the major PA observed in Queensland honey. Examination of the PAs co-occurring with lycospamine (**3**) and lycopsamine *N*-oxide (**6**), in the source plant would enable us to establish a unique floral PA fingerprint that could be correlated with PAs observed in honey. In past studies, *Echium plantagineum* L. (Paterson’s curse) has been named as the source of lycopsamine (**3**) in Australian honey [35], despite the fact lycopsamine (**3**) is usually only a minor alkaloid in *Echium* spp. [11,27,36,37]. In fact, a previous European study noted the presence of high amounts of lycopsamine (**3**) (607 ng/g) compared to low amounts of echimidine (**7**) (15 ng/g) in imported Australian honeys, and postulated an unknown plant source as a possible interpretation [27]. Indeed our analysis of *E. plantagineum* revealed that after Zn reduction echimidine (**7**) and echiumine were the dominant PAs, with both lycopsamine (**3**) and intermedine (**1**) present in much lower quantities. Clearly *E. plantagineum* is not the major source of lycopsamine (**3**) seen in our Queensland honeys, which is also consistent with the more temperate distribution of this species within Australia [38]. Other species/genera known internationally to contain lycopsamine (**3**) (and intermedine (**1**)) include *Anchusa off*., *Borago off., Lithospermum* spp., and *Symphytum* spp., and *Eupatorium* spp. [39], and are generally not geographically distributed within Australia [40]. They can logically be excluded as potential lycopsamine (**3**) floral sources.

When considering PA species which are known to be prevalent in Queensland, both *Ageratum* and *Aminscka* spp. have been reported to contain lycopsamine (**3**). *Ageratum conyzoides* for example has been reported to contain lycopsamine (**3**) and echinatine [41,42] or lycopsamine (**3**) and 3′-*O*-acetyllycopsamine [43]. A targeted screen by Avula reported lycopsamine (**3**) and its *N*-oxide (**6**) as the two major PAs, together with minor amounts of dihydrolycopsamine, dihydrolycopsamine *N*-oxide and echinatine [1,44]. The closely related *Ageratum houstonianum* is locally abundant in Queensland, and our analysis of Zn reduced plant extract revealed the predominance of retrohoustine, heliohoustine and tentatively echinatine (ratio 2.7:1.7:1 respectively), with much lower amounts of lycopsamine (**3**) and intermedine (**1**) (data not shown). This result is consistent with analysis of this same species from Mexico that showed that lycopsamine (**3**) was not the predominant pyrrolizidine alkaloid present with three other pyrrolizidine alkaloids (retrohoustine, heliohoustine and isoretrohoustine) isolated in greater amounts than lycopsamine (**3**) [45]. Lycopsamine (**3**) and intermedine (**1**) have also been identified in *Amscinckia* spp. [46], with NMR analysis revealing the relative proportion of intermedine (**1**) to lycopsamine (**3**) varied from roughly 2:1 to 1:2 in *A. intermedia, A. hispida,* and *A. lycopsoides*. *Amsinckia* spp. are however regionally controlled as noxious weeds in Australia, and not likely to be a widely abundant PA sources in Queensland. The invasive aquatic weed *Gymnocoronis spilanthoides* has been recently been shown [47] to contain predominantly lycopsamine (**3**) followed by intermedine (**1**), however, this species is also controlled by government eradication programs. None of these plant species matched either the predominant lycopsamine (**3**) profile observed in our Queensland sourced honey or the regional abundance of plant species.

Historically lycopsamine (**3**) was identified in the hair pencil of Australian danaid butterflies in Queensland in a region where *Amsinckia* plants are rare [48]. An examination of the native vines *Parsonsia straminea* (family Apocynaceae) and *Parsonsia eucalyptophylla*, by these authors revealed the presence of lycopsamine (**3**) and intermedine/indicine (**1** or **2**), and acetyl derivatives. As native *Parsonsia* species occur widely in Queensland this species was deduced as the source of lycopsamine (**3**) in danaid butterflies [49]. Lycopsamine-type PAs have been identified in a number of species in Apocynaceae [50].

Interestingly, in a study of butterfly food plants, a comparison of *Parsonsia straminea* flowers revealed the ratio of lycopsamine *N*-oxide (**6**) to intermedine *N*-oxide (**4**) to other alkaloids of 98:1:1. By contrast, *Ageratum sp.* gave a predominance of two M+ 269 isomers compared to lycopsamine (**3**) (45:48:1) [51]. Evidently, lycopsamine (**3**) and intermedine (**1**) and their *N*-oxides are present in a wide variety of plant species, but we sought to identify an origin for the almost exclusive predominance of lycopsamine (**3**) (and its *N*-oxide (**6**)) and these literature reports of *Parsonsia* provided the best clue.

### 2.7. Pyrrolizidine Alkaloids Determined in Parsonsia Vines

Local *Parsonsia straminea* (Qld Herbarium ID AQ522465) was collected and re-examined for PA content using our described HRAM LC-MS/MS method. The plant pyrrolizidine alkaloids were present primarily as the *N*-oxides (96% in the leaves and stems, 99% in the pods, 93% in the nectar and 80% in the pollen). The plant pyrrolizidine alkaloids were analysed with and without reduction by Zn to enable comparison with the honey alkaloids (primarily free alkaloids) as previously observed [25,52]. The SCX SPE methodology was previously demonstrated to be suitable for plant extracts [36]. The investigations aimed to determine for the first time whether and to what extent PAs found in honey are sourced from *Parsonsia straminea* (or closely-related *Parsonsia* species, a number of which are widespread in coastal regions of eastern Australia [53]). High resolution accurate mass (HRAM) data, combined with RT comparison with pyrrolizidine alkaloids standards enabled identification of the major pyrrolizidine alkaloids in *P. straminea* (Table 3).

In the *P. straminea* nectar, the ratio of lycopsamine (**3**) and its *N*-oxide (**6**) to intermedine (**1**) and its *N*-oxide (**4**) was >45–50:1, in the flowers it was 78:1, in anthers/pollen >50:1, in the pods it was >50:1, whilst in the leaves, ~3:1.

Minor peaks after reduction were tentatively identified by analysis of the HRAM data (Table 4) and corresponded to tessellatine (**9**) or isomer (a C7 isomer, found 300.1801, calculated for C_15_H_25_NO_5_+H^+^: 300.1805), a further C9 lycopsamine isomer (found 300.1803, calculated for C_15_H_25_NO_5_+H^+^: 300.1805), 3′-*O*-acetyllycopsamine (found 342.1905, calculated for C_17_H_28_NO_6_+H^+^: 342.1917), 3′-*O*-acetylintermedine (found 342.1924, calculated for C_17_H_28_NO_6_+H^+^: 342.1917) and two helioamplexine isomers (found 314.1958 and 314.1958, calculated for C_16_H_27_NO_5_+H^+^: 314.1962). The corresponding *N*-oxides were found in the non-reduced plant extract. Tessellatine (**9**) has the same necic acid as lycopsamine (**3**) but is esterified at the C7 necine position rather than C9 as seen in lycopsamine (**3**). The C7 esterification is evidenced in the predominant (base peak) fragment ion *m*/*z* 156.1019 (calculated for C_8_H_14_NO_2_^+^ 156.1019) characteristic of C7 monoesters [54,55], which display much smaller peaks at *m*/*z* 138.0913, 120.0809 and 94.0656 than C9 monoesters lycopsamine (**3**)/indicine (**2**)/intermedine (**1**). The diastereomeric 3′-*O*-acetyllycopsamine and 3′-*O*-acetylintermedine exhibited a similar MS breakdown to that seen in 3′-*O*-angelylindicine [25], with a base peak of *m/z* 94.0655 and other typical peaks of C9 monoesters of retronecine, 156.1019, 138.0913 and 120.0809. In these acetyl compounds, the lack of a peak at *m*/*z* 198.1125 and the lack of a base peak at *m*/*z* 214.1074 in the corresponding *N*-oxides, excluded the 7-*O*-acetyl substitution pattern [1,56]. Similarly the two helioamplexine isomers had identical MS to that seen in helioamplexine (**8**) (the C-6′ homoanalogue of indicine) [25], and these components present in *P. straminea* which did not co-elute with helioamplexine were deduced to be the corresponding C-6′ homoanalogues of lycopsamine and intermedine.

Interestingly, PAs tentatively assigned as 3′-*O*-glucosyllycopsamine (found 462.2336, calculated for C_21_H_35_NO_10_+H^+^: 462.2336) and 3′-*O*-glucosylintermedine (found 462.2335, calculated for C_21_H_35_NO_10_+H^+^: 462.2336) and the corresponding *N*-oxides (found 478.2286 and 478.2289, calculated for C_21_H_35_NO_11_+H^+^: 478.2283), were also identified in minor amounts in the pods and nectar (Table 4). The MS^2^ spectra exhibited virtually identical MS^2^ to the parent alkaloids **1** and **3**, **4** and **6**. A 3′-glucopyranosyl 2,3-dihydro-1*H*-pyrrolizin-1-one derivative has previously been reported from *Cynoglossum gansuense* [57]. Additionally, five isomeric components with MH^+^ 286.1649 were also detected in *P. straminea* reduced extracts, with MS data consistent with these being desmethyl analogues of lycopsamine, i.e ideamine A (**10**) isomers (four esterified at C9, one at C7). Ideamine A *N*-oxide has previously been found in insects feeding on *Parsonsia laevigata* leaves [58,59]. Tessellatine (**9**), 3′-*O*-acetyl- and 7-*O*-acetyllycopsamine/intermedine and their *N*-oxides have been previously identified in *Amsinckia* or *Cryptantha* species [54,55,60,61]. To positively identify the PAs in lycopsamine-rich honey samples as originating from *Parsonsia straminea*, we sought to find some of these same minor PA components of this plant in honey.

### 2.8. Honey PA Profiles Linked to P. straminea

**Figure 7:**
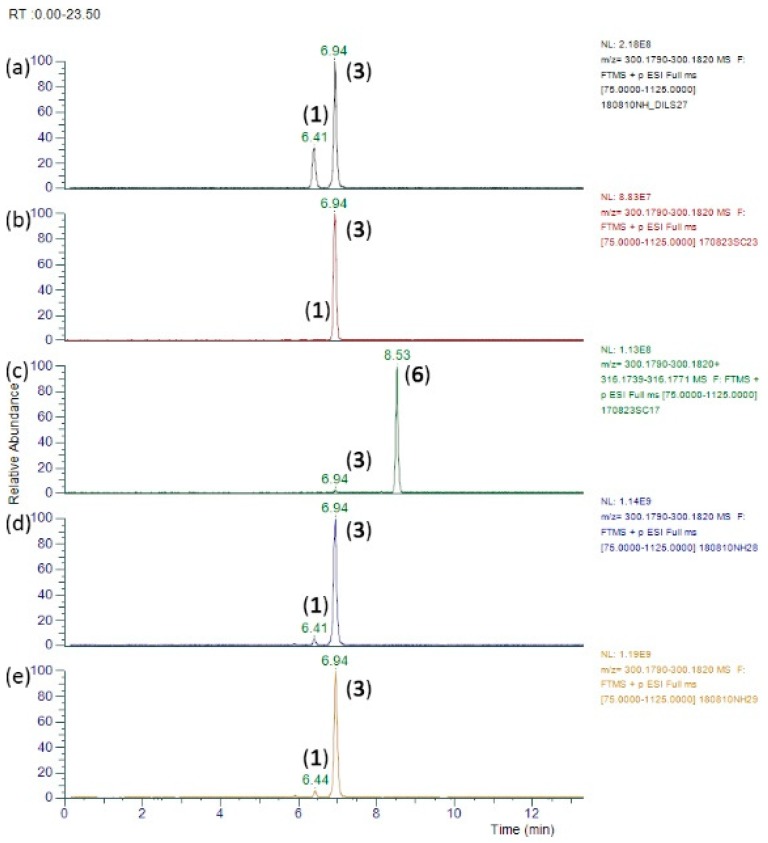
HRAM LC-MS/MS chromatograms (*m*/*z* 300.1805, *m*/*z* 316.1755) comparing the major pyrrolizidine alkaloids in *Parsonsia straminea* and honey: (**a**) intermedine (**1**) and lycopsamine (**3**) in *Parsonsia straminea* leaves (Zn reduced) (**b**) intermedine (**1**) and lycopsamine (**3**) *in Parsonsia straminea* flowers (Zn reduced) (**c**) lycopsamine (**3**) and its N-oxide (**6**) in *Parsonsia straminea* flowers (unreduced) (**d**) intermedine (**1**) and lycopsamine (**3**) in honey sample H-PA#146 (**e**) intermedine (**1**) and lycopsamine (**3**) in honey sample H-PA#157.

The detection of minor alkaloids in *Parsonsia straminea* provides a distinctive PA fingerprint in its HRAM LC-MS/MS profile, albeit in minor quantities compared to the major alkaloid lycopsamine (**3**). By comparison with the PA profile observed in market honey samples, there is clear evidence that this plant species is being used as a honey floral source by bees (Figure 7). 

**Figure 8:**
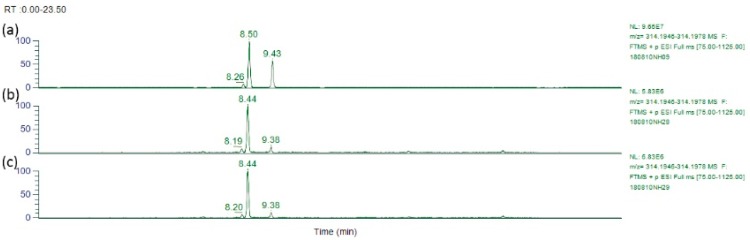
HRAM LC-MS/MS chromatograms (*m*/*z* 314.1911) comparing minor peaks in *Parsonsia straminea* and honey (**a**) isomers of helioamplexine (**8**) in *Parsonsia straminea* leaves (Zn reduced) (**b**) isomers of (**8**) in honey sample H-PA#146 (**c**) isomers of (**8**) in honey sample H-PA#157.

Honey samples such as H-PA#146 and H-PA#157 were independently purchased. When these honey samples were analysed against the 30 PA standards in our screen (Table 1), only the major alkaloid lycopsamine (**3**) and lesser intermedine (**1**) (and their *N*-oxides) were detected (Table 3). Characteristic major/minor components present in certain honeys (Table 4) included in addition to lycospamine and intermedine, the helioamplexine isomers at RT 8.20, 8.44 and 9.38 min (Figure 8) and putative 3′-*O*-acetylintermedine (8.91 min) and 3′-*O*-acetyllycospamine (9.64 min). The tentatively assigned 3′-*O*-glucosylintermedine and 3′-*O*-glucosyllycopsamine were also identified in these honey samples with the MS^2^ spectra observed identical to that found in the plant pods and nectar. Non-toxic dihydrolycopsamine isomers were also identified in the plant and honey. Due to the low levels of these minor PAs in the plant, they were seen most readily in honey samples highest in lycopsamine (**3**) (eg., H-PA#19,157,146).

### 2.9. Plant Origins of PAs in Honeys Surveyed

Of the 30 PA standards utilised in our survey, fifteen PAs (50%) were not detected in any of the market honey samples (Table 5). As shown in Table 5, based on profiles of alkaloids identified, most of the honey PAs were likely sourced from *Parsonsia straminea* or *Heliotropium amplexicaule*, with honey containing *Parsonsia* alkaloids being dominant in lycopsamine (**3**) (up to 3100 ng/g) and honey containing *Heliotropium amplexicaule* alkaloids dominant in indicine (**2**) (up to 1700 ng/g). PAs sourced from *Echium plantagineum* were much lower, with the dominant PA detected being echimidine (**7**) (up to 260 ng/g) in agreement with previous studies [11,62]. Even lower levels of PAs from *Heliotropium europaeum* (containing lasiocarpine, heliotrine and europine [11,62] (and their *N*-oxides)) and Senecio species (most likely *Senecio madagascariensis*) [63] were detected (Table 5). Of course, many of the honey samples are ascribed by their label to particular non-PA producing floral sources, so the observation of PAs in these honeys is a product of either the natural foraging of bees on different available plants, or the blending of honeys in the packaging process. This co-foraging/blending is also evident in honey samples that show co-occurrence of pyrrolizidine alkaloids from multiple floral sources, for example, honeys containing indicine (**2**) (from *H. amplexicaule*) and lycopsamine (**3**) (likely from *P. straminea* due to lack of the dominant PA echimidine (**7**) as present in *E. plantagineum*). Both these sets of PAs were present in significant levels in H-PA#11, 32, 216, 630 and 642. Geographically both the low-growing heliotrope, *H. amplexicaule*, and the arboreal vine, *P. straminea*, can co-occur in sub-tropical coastal regions of Queensland [53,64], so the co-occurrence of their respective alkaloids in honey would seem logical if both plants are visited by foraging bees within the same landscape. The high abundance of alkaloids from these quite different plant species in honey suggests that both are attractive to foraging bees, and where possible both species should be avoided when siting honey hives. It is apparent that the ‘standard set’ for PA/PANO testing of honeys varies depending on the natural flora of the region, as well as the cultivated plants present. In this study erucifoline, jacobine, monocrotaline, senciphylline, or their corresponding *N*-oxides and senkirkine or trichodesmine were not found in the honey tested, which is a considerably different result to those found recently in Schleswig-Holstein region of Germany [28].

### 2.10. Honey as a Dietary Source of Pyrrolizidine Alkaloids

Major supermarket honeys by comparison represent blended honeys from diverse locations, some of which attributed the specific floral source and in general contained only low levels of PAs. It has been observed previously that blended retail honeys had a lower PA content, but that PAs were present in more samples [65]. In this study, for supermarket honeys (*n* = 129), PAs were detected in 84% of honeys, and showed highest total PA levels of 1400 ng/g. For supermarket honeys, the mean total PA level of PA-positive samples was 120 ng/g and the median level was 61 ng/g.

Certain small producer honeys displayed the highest levels of pyrrolizidine alkaloids, with the PA content dependent on the location and attractiveness of PA containing plants to foraging honey bees. Paradoxically, even though analysed PA content of small producer honeys range from <LOR to an alarming 3000 ng/g, if equal amounts of each of these 205 small producer honeys were blended, the hypothetical resultant mixed honey would have a PA content of only 240 ng/g (i.e., the average PA content of all of these 465 honeys).

It has been observed previously in South American honeys that raw honeys showed greatest variety due to the availability of PA containing plants near to hives [65].

The cumulative toxicity of the 1,2-unsaturated PAs have been demonstrated in animal studies and genotoxicities/tumorigenicities were induced by hepatic metabolism of PAs [66]. Consequently, provisional tolerable daily intakes (PTDI) have been recommended to control the human consumption of PAs [5,10,39,67].

Using the Australian FSANZ provisional tolerable daily intake (PTDI) of 1 μg/kg BW/day, 0% of honeys tested (total *n* = 465) exceeded the limit for a 70 kg adult consuming 20 g of honey per day, but 19% of honeys tested exceeded the limit for a 15 kg child consuming 50 g of honey per day. Applying the lowest recommended PTDI (EFSA, COT, BfR) of 0.007 μg/kg BW/day, 63% of honeys tested exceeded the limit for a 70 kg adult consuming 20 g of honey per day and 84% of honeys tested exceeded the limit for a 15 kg child consuming 50 g of honey per day.

The PA content of honey samples varies with geographical location and climate, determined by the type and distribution of PA containing plants and by the propensity for bees to forage on these plants [65,68]. Lycopsamine (**3**) and intermedine (**1**) are present in many PA-producing plants, with the knowledge of the plants distributed in Australia and the ratio to other PAs present, it is likely that *Parsonsia straminea* is a major contributor to the high PA levels observed in certain honeys in this study. Of course, it is possible that there is more than one PA source of lycopsamine (**3**), with a small portion of lycopsamine contamination of honey potentially originating from *Echium plantagineum* and *Ageratum houstonianium*. Also, there are likely other PA containing plants that have not been considered. It is also possible that not all PAs present in honey have been identified by comparison with standards and by analysis of the top MSMS. Despite the observation that of the PAs tested in experimental rats, lycopsamine (**3**) induced the lowest levels of liver DNA adducts (formed from PA derived reactive pyrrolic metabolites), PA containing plants are the most common poisonous/carcinogenic plants affecting livestock, wildlife and humans [69]. Beekeepers are advised to avoid these known plant genera around the hive/apiary as much as possible to reduce PA contamination in honey.

## 3. Conclusions

The HRAM LC-MS/MS method for pyrrolizidine alkaloid analysis described here enables the ready resolution of isomeric alkaloids of the lycopsamine-type. The described simple adjustment of column conditions to a lower temperature was effective in resolving the problematic pairs of indicine/lycopsamine alkaloids present in Australian honey. This resolution has enabled us to identify *Parsonsia* vines as a previously unsuspected source of PA contamination in Australian honey. Low temperature chromatographic resolution may have as yet unexplored application in resolving other similar diastereomeric pyrrolizidine alkaloid isomers, of which there are many within the known pyrrolizidine alkaloids, many of which do not have commercially available standards.

## 4. Materials and Methods

### 4.1. Chemicals and Solvents

In total, 30 pyrrolizidine alkaloid standards were utilized in a high resolution accurate mass (HRAM) LC-MS/MS screen. Echimidine, erucifoline, europine, heliotrine, indicine, intermedine, jacobine, lasiocarpine lycopsamine, monocrotaline, retrorsine senecionine, seneciphylline, senecivernine, and their respective *N*-oxides, were purchased together with senkirkine and trichodesmine from Phytolab GmbH & Co. KG (Vestenbergsgreuth, Germany) and had a purity >89%. All other chemicals and solvents were of analytical reagent or HPLC grade purity. Water used for sample preparation and HPLC was Milli-Q purified (Merck Millipore, Darmstadt, Germany).

### 4.2. Honey Samples

Honey samples (465 in total) were purchased between September 2016 and December 2017 directly from Queensland supermarkets, fruit shops, local markets, and producers.

### 4.3. Honey Alkaloid Extraction

Honey samples (1 g) were dissolved in aqueous H_2_SO_4_ (0.05 M, 10 mL) centrifuged and the supernatant applied to preconditioned Agilent SPE Bond Elut 100 mg LRC-SCX columns (Agilent Technologies, Folsom, CA, USA). SPE cartridges were washed with water (10 mL) and methanol (10 mL), and pyrrolizidine alkaloids were then eluted with 3% ammonia in methanol (3 mL). The eluate was evaporated to dryness under nitrogen, and the residue reconstituted in 5% methanol in water (1 mL) for HRAM LC-MS/MS analysis.

### 4.4. Honey Method Validation

The validation of the method was conducted according to the National Association of Testing Authorities (NATA) guidance document [22]. The method was validated in 3 blank honeys, and based on results for 10 spiked samples at a spiking level of 5 ng/g (Table 2), 10 blank samples and 10 non-extracted spike samples and the recoveries determined. Limit of detection (LOD) was calculated as 3*s*. Limit of quantitation was calculated as 9*s*. Limit of reporting was set at the levels the samples were spiked, also the level of the lowest standard used for the calibration curve. The uncertainties given are at the 95% confidence level as required by the NATA [22,23]. Replicate samples were prepared for every tenth honey test sample to assess reproducibility. The difference between replicate samples (coefficient of variance %) was typically 0.12–6.7%. High samples were diluted to levels within the calibration curve and re-run. SPE wash steps and further elutions with 3% ammonia in methanol (3 mL) were analysed for residual PAs and the extraction was found to be exhaustive. Table 2 shows good recoveries for most PAs.

### 4.5. Plant Alkaloid Extraction

#### 4.5.1. Plant Source

*Parsonsia straminea* was collected from a suburban area in the south of Brisbane and was taxonomically identified by the Queensland Herbarium, with a voucher specimen (AQ522465) incorporated into their collection. The *Parsonsia straminea* foliage sample was a collection of stems and leaves, and was freeze dried, milled and stored frozen prior to analysis. Pods were collected separately and freeze-dried, milled and frozen. Flowers were sampled as both intact flowers (freeze-dried, milled and frozen) or utilized to provide nectar and pollen separately. Nectar was separated from flowers using a microcap capillary, and anthers and pollen were separated from other flower plants with tweezers and desiccated.

#### 4.5.2. Foliage and Seed Pod Extracts

Dried milled plant leaves and stems (1 g) and seed pods (1 g) were separately dissolved in methanol (10 mL), vortexed (20 s), shaken (30 min) then centrifuged (4800 rpm, 10 min) and the supernatants removed and concentrated to dryness under nitrogen. The residues were dissolved in aqueous H_2_SO_4_ (0.05 M, 10 mL) centrifuged (4800 rpm, 10 min) and a portion of the supernatants (0.1 mL) were applied to preconditioned Agilent SPE Bond Elut 500 mg LRC-SCX columns. SPE cartridges were washed with water (10 mL) and methanol (10 mL), and pyrrolizidine alkaloids were then eluted with 3% ammonia in methanol (10 mL). The eluate was evaporated to dryness under nitrogen, and the residue reconstituted in 5% methanol in water (1 mL) for HRAM LC-MS/MS analysis.

#### 4.5.3. Whole Flower Extracts

Dried and milled flowers (0.1 g) were dissolved in methanol (2 mL), vortexed (20 s), shaken (30 min) then centrifuged (4800 rpm, 10 min) and the supernatant removed and concentrated to dryness under nitrogen. The residue was dissolved in aqueous H_2_SO_4_ (0.05 M, 1 mL), centrifuged (4800 rpm, 10 min) and, for each sample, a portion of the supernatant (0.1 mL) was applied to a preconditioned Agilent SPE Bond Elut 500 mg LRC-SCX column. Each SPE cartridge was washed with water (10 mL) and methanol (10 mL), and pyrrolizidine alkaloids were then eluted with 3% ammonia in methanol (10 mL). The eluate was evaporated to dryness under nitrogen, and the residue reconstituted in 5% methanol in water (1 mL) for HRAM LC-MS/MS analysis.

#### 4.5.4. Zinc Reduced Extracts

Another portion of each aqueous H_2_SO_4_ supernatant (0.5 mL) derived from leaves/stems, pods and flowers was treated with Zn dust (100 mg) and stirred (2 h). After centrifugation (4800 rpm, 10 min), a portion of the supernatants (0.1 mL) was applied to a preconditioned Agilent SPE Bond Elut 500 mg LRC-SCX column. Each SPE cartridge was washed with water (10 mL) and methanol (10 mL), and pyrrolizidine alkaloids were then eluted with 3% ammonia in methanol (10 mL). The eluate was evaporated to dryness under nitrogen, and the residue reconstituted in 5% methanol in water (1 mL) for HRAM LC-MS/MS analysis.

#### 4.5.5. Floral Nectar Extract

Nectar (22.5 mg) was obtained from fresh flowers using a microcap capillary, dissolved in MeOH (0.5 mL) and diluted 1 in 100 with 5% methanol in water (1 mL) for HRAM LC-MS/MS analysis.

#### 4.5.6. Pollen Extract

Dessicated anthers and pollen were placed in hexane and shaken (1 min). The hexane containing pollen was separated from the anthers and evaporated to dryness under nitrogen. The resulting pollen (0.36 mg) was dissolved in 5% methanol in water and diluted as required for LCMS/MS analysis.

### 4.6. HRAM LC-MS/MS Analysis

Samples were analysed using a Vanquish UHPLC in combination with Q Exactive Orbitrap high resolution accurate mass (HRAM) spectrometry system (Thermo Fisher Scientific, Bremen, Germany). LC-MS/MS separation was achieved on a Kinetex XB-C18 analytical column (100 × 2.1 mm, 2.6 µm, 100 Å) at 5 °C. Analysis conditions: binary solvent system, solvent A (ammonium formate (5 mM) and formic acid (0.1%) and solvent B (95% *v*/*v* methanol/water with ammonium formate (5 mM) and formic acid (0.1%)). Compounds were eluted from the column at 0.3 mL min^−1^ with mobile phase B held at 5% for 3 min followed by linear gradients of B from 5–50% (3–15 min), 50–80% (15–18.5 min), 80–100% (18.5–19 min), where it was held for 30 sec, before reducing from 100–5% over 6 sec, where it was held until stop at 23.5 min. Instrument control, data acquisition and analysis were conducted using Tracefinder 4.1 from Thermo Fisher Scientific. Alkaloid detection was performed by positive electrospray ionisation (ESI) with a spray voltage of 3500 V and a vaporiser temperature of 400 °C. MS analysis run with arbitrary pressures of sheath gas 48, aux gas 11, sweep gas 2, spray voltage 3.5 kV, capillary temperature of 320 °C, auxiliary gas heater at 350 °C and used full scan/dd-MS^2^ mode. Full scans were conducted at a resolution of 70,000 FWHM (at *m*/*z* 200), with an AGC target of 1.00 × 10^6^. The maximum time of accumulating ions per scan event was 10 ms with a scan range of 75–1125 *m*/*z*. Data dependent acquisition (dd-MS^2^) was conducted at a resolution of 17,500 with an AGC target of 1.00 × 10^6^. The maximum time of accumulating ions per scan event was 50 ms. Normalized collision energy (nce) was set to 50% and an isolation window of 1.0 *m*/*z* was utilized. Dynamic exclusion was set to 3 s preventing subsequent triggering of the same ion in data dependent scans. A maximum of 5 most abundant precursors could be selected for dd-MS^2^ per scan event.

Pyrrolizidine alkaloid levels in honey/plant material were quantitated against certified PA standards, with calibration curves obtained for each of the 30 pyrrolizidine alkaloid standards injected at 5, 10, 20, 50 and 100 and 200 ppb (in duplicate/triplicate). Honey or plant extracts were analysed by HRAM LC-MS/MS to detect pyrrolizidine alkaloids and their *N*-oxides by matching of retention time with the corresponding standard and identified by their precursor parent ion (M+H^+^) and confirmed by the detection of product ions (Table 1). The identity of these and further alkaloids was assigned by use of the high resolution accurate mass data provided by the Q Exactive mass spectrometer, enabling the determination of elemental composition of parent and fragment ions (Table 3 and Table 4).

## Figures and Tables

**Table 1:** Details of pyrrolizidine alkaloids used in the Orbitrap analysis of PA containing plants and honey, including formulae, retention times, precursor ions used for quantitation and confirmatory product ions.

Compound	Formula	Average R_T_ (min)	Precursor Ion (MH^+^)	Product Ions					
Echimidine (**7**)	C_20_H_31_NO_7_	12.86	398.2173	120.0809	83.0497	55.0550			
Echimidine *N*-oxide	C_20_H_31_NO_8_	12.80	414.2122	396.2004	352.1745	254.1379	220.1326	137.0833	
Erucifoline	C_18_H_23_NO_6_	6.23	350.1598	322.1642	220.1329	164.1066	138.0911	120.0807	
Erucifoline *N*-oxide	C_18_H_23_NO_7_	8.03	366.1547	278.1386	218.1172	164.1067	136.0756	119.0729	
Europine	C_16_H_27_NO_6_	6.97	330.1911	254.1385	156.1019	138.0914	120.0810	96.0812	
Europine *N*-oxide	C_16_H_27_NO_7_	7.86	346.1860	328.1743	270.1328	256.1172	172.0964	155.0937	
Helioamplexine	C_16_H_27_NO_5_	9.21	314.1962	156.1017	138.0913	120.0808	94.0655		
Helioamplexine *N*-oxide	C_16_H_27_NO_6_	10.42	330.1911	172.0966	155.0938	138.0913	111.0913	94.0653	
Heliotrine	C_16_H_27_NO_5_	9.56	314.1962	156.1017	138.0913	120.0808	94.0655		
Heliotrine *N*-oxide	C_16_H_27_NO_6_	10.46	330.1911	172.0966	155.0938	138.0913	111.0913	94.0653	
Indicine (**2**)	C_15_H_25_NO_5_	6.67	300.1806	156.1019	138.0914	120.0810	94.0656	82.0657	
Indicine *N*-oxide (**5**) and intermedine *N*-oxide (**4**) (n.r.) ^a^	C_15_H_25_NO_6_	8.20	316.1755	226.1437	172.0968	155.0941	138.0914	111.0682	94.0656
Intermedine (**1**)	C_15_H_25_NO_5_	6.26	300.1806	210.1488	156.1019	138.0914	120.0810	94.0656	
Jacobine	C_18_H_25_NO_6_	6.56	352.1755	308.1485	280.1539	262.1432	234.1483	155.1063	
Jacobine *N*-oxide	C_18_H_25_NO_7_	7.91	368.1704	296.1485	190.1222	139.0989	121.0885	120.0807	
Lasiocarpine	C_21_H_33_NO_7_	14.92	412.2330	238.1435	156.1020	138.0914	120.0810	94.0656	
Lasiocarpine *N*-oxide	C_21_H_33_NO_8_	16.14	428.2279	410.2168	352.1746	328.1753	254.1384	220.1333	137.0835
Lycopsamine (**3**)	C_15_H_25_NO_5_	6.80	300.1806	156.1017	138.0914	120.0808	94.0655		
Lycopsamine *N*-oxide (**6**)	C_15_H_25_NO_6_	8.65	316.1755	172.0964	155.0937	138.0911	136.0755	94.0654	
Monocrotaline	C_16_H_23_NO_6_	2.88	326.1598	280.1548	237.1354				
Monocrotaline *N*-oxide	C_16_H_23_NO_7_	7.19	342.1547	314.1590	296.1487	236.1274	137.0833	119.0729	
Retrorsine	C_18_H_25_NO_6_	9.13	352.1755	324.1802	138.0913	120.0808	94.0655		
Retrorsine *N*-oxide	C_18_H_25_NO_7_	9.64	368.1704	220.1340	154.0862				
Senecionine	C_18_H_25_NO_5_	11.13	336.1806	308.1864	120.0809				
Senecionine *N*-oxide	C_18_H_25_NO_6_	11.83	352.1755	324.1825	220.1332				
Seneciphylline	C_18_H_23_NO_5_	9.52	334.1649	306.1706	120.0811				
Seneciphylline *N*-oxide	C_18_H_23_NO_6_	10.46	350.1598	322.1656	246.1495				
Senecivernine	C_18_H_25_NO_5_	10.84	336.1806	308.1848	153.0907	138.0911	120.0807	94.0654	
Senecivernine *N*-oxide	C_18_H_25_NO_6_	11.45	352.1755	324.1795	220.1327	154.0859	136.0755	120.0807	
Senkirkine	C_19_H_27_NO_6_	13.62	366.1911	168.1020	150.0915				
Trichodesmine	C_18_H_27_NO_6_	8.79	354.1911	308.1857	223.1203	222.1489	164.1071	121.0889	

^a^ n.r. = not resolved.

**Table 2:** Method validation results for pyrrolizidine alkaloids in honey.

Display Name	Spiking Level (ng/g)	LOD (3*s*)	LOQ (9*s*)	LOR	Units	Recovery (%)	Calculated Standard Uncertainty (% RSD)
Echimidine (**7**)	5	0.7	2	5	ng/g	88	11
Echimidine *N*-oxide	5	0.5	2	5	ng/g	60	15
Erucifoline	5	0.7	2	5	ng/g	34	26
Erucifoline *N*-oxide	5	0.5	2	5	ng/g	26	31
Europine	5	1	2	5	ng/g	98	16
Europine *N*-oxide	5	0.5	1	5	ng/g	94	6
Heliotrine	5	0.5	0.7	5	ng/g	108	4
Heliotrine *N*-oxide	5	0.2	0.5	5	ng/g	83	2
Indicine	5	0.5	2	5	ng/g	98	8
Indicine *N*-oxide (**5**) and intermedine *N*-oxide (**4**) as indicine *N*-oxide (**5**)	5	0.5	1	5	ng/g	78	7
Intermedine (**1**)	5	0.5	2	5	ng/g	94	8
Jacobine	5	2	5	5	ng/g	61	30
Jacobine *N*-oxide	5	1	5	5	ng/g	36	51
Lasiocarpine	5	0.5	1	5	ng/g	82	6
Lasiocarpine *N*-oxide	5	0.5	0.7	5	ng/g	79	6
Lycopsamine (**3**)	5	0.2	0.7	5	ng/g	95	4
Lycopsamine *N*-oxide (**6**)	5	0.7	2	5	ng/g	81	14
Monocrotaline	5	2	5	5	ng/g	89	24
Monocrotaline *N*-oxide	5	0.7	2	5	ng/g	89	13
Retrorsine	5	1	2	5	ng/g	96	13
Retrorsine *N*-oxide	5	0.7	2	5	ng/g	58	22
Senecionine	5	0.7	2	5	ng/g	90	11
Senecionine *N*-oxide	5	0.5	2	5	ng/g	58	16
Seneciphylline	5	0.7	2	5	ng/g	70	15
Seneciphylline *N*-oxide	5	0.5	1	5	ng/g	16	36
Senecivernine	5	0.5	2	5	ng/g	86	9
Senecivernine *N*-oxide	5	0.7	2	5	ng/g	53	20
Senkirkine	5	0.5	0.7	5	ng/g	91	5
Trichodesmine	5	3	7	7	ng/g	78	69

**Table 3:** High resolution accurate mass (HRAM) data for pyrrolizidine alkaloids in *P. straminea* identified by comparison with PA standards.

Alkaloid	Typical R_T_ (min)	Molecular Ion Formula	Calculated [M+H]^+^	Observed [M+H]^+^	Mass Spectral Data (Rel. Int. %)
Intermedine (**1**)	6.27	[C_15_H_25_NO_5_ + H] ^+^	300.1805	300.1804	300.1804 (6) 256.1539 (1) 156.1019 (53) 139.0992 (11) 138.0914 (31) 120.0809 (18) 112.0759 (2) 108.0811 (2) 96.0812 (4) 95.0733 (4) 94.0656 (100) 82.0657 (4)
Lycopsamine (**3**)	6.80	[C_15_H_25_NO_5_ + H] ^+^	300.1805	300.1805	300.1805 (9) 256.1539 (0.3) 156.1020 (59) 139.0993 (13) 138.0915 (31) 120.0811 (19) 112.0760 (3) 108.0813 (1) 96.0813 (5) 95.0734 (5) 94.0656 (100) 82.0657 (6)
Intermedine *N*-oxide (**4**)	8.19	[C_15_H_25_NO_6_ + H] ^+^	316.1755	316.1752	316.1752 (41) 298.1631 (1) 272.1491 (2) 172.0967 (100) 155.0940 (18) 154.0862 (8) 138.0914 (55) 137.0837 (6) 136.0757 (15) 112.0759 (5) 111.0682 (12) 94.0655 (19) 93.0577 (13) 82.0419 (3)
Lycopsamine *N*-oxide (**6**)	8.64	[C_15_H_25_NO_6_ + H] ^+^	316.1755	316.1752	316.1752 (46) 298.1653 (1) 272.1491 (2) 172.0967 (100) 155.0940 (17) 154.0862 (10) 138.0913 (63) 137.0835 (6) 136.0758 (17) 112.0759 (5) 111.0681 (13) 94.0655 (22) 93.0577 (16) 82.0419 (4)

**Table 4:** High resolution accurate mass (HRAM) data for pyrrolizidine alkaloids in *P. straminea* identified by analysis of fragmentation patterns, together with those identified in honey samples high in lycopsamine (identification in honey indicated by *).

Alkaloid	Typical R_T_ (min)	Molecular Ion Formula	Calculated [M+H]^+^	Observed [M+H]^+^	Mass Spectral Data (Rel. Int. %)
* Tessellatine (**9**) or isomer	6.34	[C_15_H_25_NO_5_ + H] ^+^	300.1805	300.1801	300.1801 (5) 156.1019 (100) 139.0990 (1) 138.0913 (6) 120.0809 (2) 112.0759 (4) 108.0810 (6) 96.0811 (1) 94.0656 (4) 82.0655 (1)
Lycopsamine isomer (isomer of **3**)	5.91	[C_15_H_25_NO_5_ + H] ^+^	300.1805	300.1803	300.1803 (7) 210.1485 (1) 156.1019 (42) 139.0992 (14) 138.0914 (26) 120.0809 (18) 112.0759 (2) 108.0812 (2) 96.0813 (5) 95.0734 (6) 94.0656 (100) 82.0657 (5)
* Helioamplexine isomer I (isomer of **8**)	8.51	[C_16_H_27_NO_5_ + H] ^+^	314.1962	314.1958	314.1958 (7) 270.1704 (0.5) 224.1643 (2) 156.1019 (44) 139.0992 (19) 138.0914 (25) 120.0810 (18) 112.0759 (2) 96.0812 (5) 95.0734 (8) 94.0656 (100) 82.0657 (7)
* Helioamplexine isomer II (isomer of **8**)	9.30	[C_16_H_27_NO_5_ + H] ^+^	314.1962	314.1958	314.1960 (5) 270.1698 (0.5) 224.1643 (2) 156.1019 (33) 139.0992 (14) 138.0914 (25) 120.0809 (18) 112.0759 (2) 96.0812 (4) 95.0733 (7) 94.0656 (100) 82.0657 (6)
* Helioamplexine isomer (C7-isomer of **8**)	8.34	[C_16_H_27_NO_5_ + H] ^+^	314.1962	314.1959	314.1959 (4) 156.1019 (100) 139.0992 (2) 138.0914 (6) 126.0916 (1) 120.0808 (2) 112.0759 (5) 108.0810 (10) 94.0655 (1) 86.0605 (2) 82.0656 (1) 80.0500 (1)
* 3′-*O*-Acetylintermedine	8.91	[C_17_H_27_NO_6_ + H] ^+^	342.1911	342.1924	342.1924 (1) 282.1700 (4) 156.1019 (16) 139.0991 (7) 138.0913 (17) 120.0809 (20) 108.0812 (1) 96.0812 (2) 96.0450 (2) 95.0734 (3) 94.0655 (100) 82.0657 (3)
* 3′-*O*-Acetyllycopsamine	9.64	[C_17_H_27_NO_6_ + H] ^+^	342.1911	342.1905	342.1905 (1) 282.1703 (2) 156.1019 (14) 139.0992 (3) 138.0913 (19) 120.0809 (19) 108.0812 (1) 96.0812 (2) 96.0448 (1) 95.0734 (2) 94.0655 (100) 82.0657 (3) 67.0549 (1)
Ideamine A (**10**) isomer I	2.43	[C_14_H_23_NO_5_ + H] ^+^	286.1649	286.1648	286.1648 (11) 242.1392 (1) 196.1335 (2) 156.1019 (43) 139.0992 (11) 138.0914 (26) 120.0809 (18) 112.0758 (2) 108.0810 (2) 96.0813 (7) 95.0734 (5) 94.0656 (100) 82.0657 (5)
Ideamine A (**10**) isomer II	3.70	[C_14_H_23_NO_5_ + H] ^+^	286.1649	286.1649	286.1649 (8) 156.1019 (35) 139.0992 (8) 138.0913 (30) 120.0808 (18) 96.0813 (4) 95.0734 (4) 94.0655 (100) 94.0593 (1) 82.0657 (3) 80.0500 (1) 67.0547 (1)
Ideamine A (**10**) isomer III	4.37	[C_14_H_23_NO_5_ + H] ^+^	286.1649	286.1649	286.1649 (0) 156.1018 (46) 139.0992 (7) 138.0913 (26) 120.0807 (17) 98.2419 (3) 95.0733 (3) 94.0656 (100) 93.0705 (3) 82.0655 (4) 67.0549 (4)
Ideamine A (**10**) isomer IV	5.10	[C_14_H_23_NO_5_ + H] ^+^	286.1649	286.1651	286.1651 (16) 258.9584 (3) 190.8082 (4) 188.1787 (3) 158.5305 (3) 156.1016 (48) 138.0913 (29) 124.0405 (3) 120.0807 (18) 94.0655 (100)
Ideamine A C7 isomer	1.72	[C_14_H_23_NO_5_ + H] ^+^	286.1649	286.1656	286.1656 (10) 156.1019 (100) 138.0915 (5) 124.0757 (2) 120.0813 (1) 112.0760 (5) 108.0811 (7) 106.0656 (4) 94.0651 (2) 86.0603 (1)
Lycopsamine *N*-oxide isomer	7.61	[C_15_H_25_NO_6_ + H] ^+^	316.1755	316.1751	316.1754 (46) 172.0967 (100) 155.0940 (21) 154.0862 (13) 138.0914 (94) 137.0836 (8) 136.0757 (25) 112.0759 (8) 111.0681 (14) 108.0811 (6) 94.0656 (35) 93.0577 (24)
7-Isomer of lycopsamine *N*-oxide	7.19	[C_15_H_25_NO_6_ + H] ^+^	316.1755	316.1751	316.1751 (5) 172.0968 (100) 155.0940 (6) 154.0863 (2) 138.0914 (3) 137.0836 (3) 136.0757 (2) 111.0681 (11) 106.0654 (6) 102.0555 (2) 94.0657 (1)
Helioamplexine *N*-oxide isomer I	9.87	[C_16_H_27_NO_6_ + H] ^+^	330.1911	330.1908	330.1908 (39) 286.1642 (4) 240.1594 (7) 172.0967 (100) 155.0940 (30) 154.0862 (11) 138.0914 (84) 136.0758 (22) 112.0760 (8) 111.0681 (18) 94.0655 (36) 93.0577 (20) 82.0419 (9)
Helioamplexine *N*-oxide isomer II	10.63	[C_16_H_27_NO_6_ + H] ^+^	330.1911	330.1909	330.1909 (46) 172.0967 (100) 155.0940 (30) 154.0862 (12) 138.0914 (91) 137.0835 (9) 136.0757 (23) 112.0759 (8) 111.0681 (20) 94.0655 (38) 93.0577 (26) 82.0419 (11)
3′-*O*-Acetyllycopsamine *N*-oxide isomer I	10.25	[C_17_H_27_NO_7_+ H] ^+^	358.1860	358.1856	358.1856 (15) 316.1758 (4) 298.1649 (40) 172.0968 (100) 155.0941 (23) 154.0863 (10) 138.0914 (73) 136.0758 (21) 111.0682 (19) 94.0656 (33) 93.0577 (21) 89.0602 (19) 87.0446 (14)
3′-*O*-Acetyllycopsamine *N*-oxide isomer II	10.92	[C_17_H_27_NO_7_+ H] ^+^	358.1860	358.1859	358.1859 (19) 316.1748 (4) 298.1648 (39) 172.0967 (100) 155.0940 (19) 154.0862 (14) 138.0914 (89) 137.0835 (8) 136.0758 (27) 112.0759 (7) 111.0681 (16) 94.0656 (44) 93.0577 (31)
7-*O*-Acetyllycopsamine *N*-oxide	10.03	[C_17_H_27_NO_7_+ H] ^+^	358.1860	358.1857	358.1857 (21) 214.1073 (100) 180.1019 (48) 178.0863 (14) 137.0836 (53) 136.0758 (17) 120.0810 (16) 119.0731 (14) 106.0654 (13) 101.0601 (25) 89.0602 (15) 73.0291 (20)
Ideamine A *N*-oxide isomer I	4.87	[C_14_H_23_NO_6_+ H] ^+^	302.1598	302.1595	302.1595 (67) 212.1275 (4) 172.0966 (91) 155.0939 (18) 154.0864 (10) 138.0913 (100) 137.0834 (7) 136.0757 (20) 112.0759 (7) 111.0681 (11) 108.0810 (6) 94.0655 (31) 93.0577 (23)
Ideamine A *N*-oxide isomer II	6.27	[C_14_H_23_NO_6_+ H] ^+^	302.1598	302.1596	302.1596 (65) 172.0968 (96) 158.1181 (7) 155.0941 (18) 154.0861 (12) 138.0913 (100) 137.0835 (10) 136.0757 (26) 112.0759 (9) 111.0680 (9) 94.0656 (31) 93.0577 (24)
* 3′-*O*-Glucosyllycopsamine	8.94	[C_21_H_35_NO_10_ + H] ^+^	462.2336	462.2336	300.1806 (12) 156.1021 (16) 139.0994 (4) 138.0915 (40) 120.0810 (16) 97.0289 (3) 96.0813 (2) 96.0451 (2) 94.0657 (100) 85.0290 (7)
* 3′-*O*-Glucosylintermedine	7.41	[C_21_H_35_NO_10_ + H] ^+^	462.2336	462.2335	300.1807 (17) 156.1019 (20) 139.0993 (5) 138.0914 (48) 120.0810 (19) 97.0289 (4) 96.0813 (2) 94.0656 (100) 91.0580 (2) 85.0290 (10)
* 3′-*O*-Glucosyllycopsamine *N*-oxide	9.89	[C_21_H_35_NO_11_ + H] ^+^	478.2283	478.2286	316.1754 (100) 172.0968 (90) 155.0941 (15) 154.0860 (10) 138.0914 (74) 137.0837 (6) 136.0758 (19) 120.0809 (6) 112.0762 (5) 111.0680 (7) 94.0657 (47) 93.0578 (12)
* 3′-*O*-Glucosylintermedine *N*-oxide	8.58	[C_21_H_35_NO_11_ + H] ^+^	478.2283	478.2289	316.1758 (100) 172.0968 (76) 155.0943 (13) 154.0863 (9) 138.0916 (56) 137.0838 (7) 136.0757 (15) 120.0808 (7) 111.0681 (7) 94.0656 (29) 93.0578 (12)

**Table 5:** Number of PA positive samples and PA concentration in honey samples (*n* = 465), grouped by potential source of PA plant origin (mean and median are for positive samples only).

Potential Plant Source of PAs in Honey	Pyrrolizidine Alkaloids	Number of Honey Samples	PA Content (ng/g)		
			Mean	Median	Max
***Parsonsia straminea*** (Monkey rope)					
	Lycopsamine	274	210	33	3100
	Lycopsamine *N*-oxide	31	320	51	1900
	Intermedine	217 ^b^	32	18	290
	Intermedine *N*-oxide ^a^	30 ^c^	86	21	660
***Heliotropium amplexicaule*** (Blue heliotrope)					
	Indicine	221	120	31	1700
	Indicine *N*-oxide/intermedine *N*-oxide (n.r.) ^a^	30 ^c^	86	21	660
	Intermedine	217 ^b^	32	18	290
	Helioamplexine ^d^	78	23	13	130
	Helioamplexine *N*-oxide ^e^	5	28	35	51
***Echium plantagineum*** (Paterson’s curse)					
	Echimidine	93	36	20	260
	Lycopsamine Intermedine	76 ^f^ 37 ^g^			
***Heliotropium europaeum*** (Potato weed)					
	Europine	26	30	11	160
	Europine *N*-oxide	4	11	7	24
	Heliotrine	17	23	15	75
	Heliotrine *N*-oxide	1	9	9	9
	Lasiocarpine	10	20	14	59
***Senecio* species**					
	Senecivernine	19	24	14	150
	Senecionine	7	13	8	41
	Retrorsine	18	20	12	91
**No occurrence/Below LOR**					
	Echimidine *N*-oxide	0			
	Erucifoline	0			
	Erucifoline *N*-oxide	0			
	Jacobine	0			
	Jacobine *N*-oxide	0			
	Lasiocarpine *N*-oxide	0			
	Monocrotaline	0			
	Monocrotaline *N*-oxide	0			
	Retrorsine *N*-oxide	0			
	Senecionine *N*-oxide	0			
	Seneciphylline	0			
	Seneciphylline *N*-oxide	0			
	Senecivernine *N*-oxide	0			
	Senkirkine	0			
	Trichodesmine	0			

^a^ Not resolved–indicine *N*-oxide and intermedine *N*-oxide co-eluted. ^b,c^ Intermedine and intermedine *N*-oxide are present in multiple plants, and prominent in both *Parsonsia straminea* and *Heliotropium amplexicaule.* ^d^ Helioamplexine was quantified using heliotrine standard curve. ^e^ Helioamplexine *N*-oxide was quantified using heliotrine *N*-oxide standard curve. ^f^ Lycopsamine observed in honey containing echimidine not necessarily attributed solely to *E. plantagineum*, but of the 93 honey samples containing echimidine, the concentration of lycopsamine was lower than echimidine in 76 honeys, which is consistent with the relative amounts observed in *E. plantagineum*. In the other 17 honeys which contain echimidine, it is likely that there is more than one source of lycopsamine. ^g^ Of the 76 honeys in which lycopsamine was at a lower level than echimidine, 37 honey samples also contained intermedine.
